# Construction of Quasi-Ordered Metal-Organic Frameworks Superstructures via Colloidal Assembly of Anisotropic Particles for Selective Organic Vapor Sensing

**DOI:** 10.3390/nano13192733

**Published:** 2023-10-09

**Authors:** Yuheng He, Ling Bai, Baocang Liu, Hongwei Duan, Jun Zhang

**Affiliations:** 1 School of Chemistry and Chemical Engineering, Inner Mongolia Engineering and Technology Research Center for Catalytic Conversion and Utilization of Carbon Resource Molecules, Inner Mongolia University, 49 Xilinguole South Road, Hohhot 010020, China; heyuheng910@163.com (Y.H.); cebcliu@imu.edu.cn (B.L.); 2 School of Materials Science and Engineering, Jiangsu University, 301 Xuefu Road, Zhenjiang 212013, China; lingmubai@ujs.edu.cn; 3 School of Chemistry Chemical Engineering and Biotechnology, Nanyang Technological University, 70 Nanyang Drive, Singapore 637457, Singapore; 4 School of Chemistry and Environmental Science, Inner Mongolia Normal University, 81 Zhaowuda Road, Hohhot 010022, China

**Keywords:** colloidal assembly, polyhedral nanoparticles, metal organic frameworks, vapor sensors

## Abstract

Colloidal assembly of anisotropic particles holds great promise for achieving diverse packing geometries and unique photonic properties. One intriguing candidate for anisotropic self-assembly is colloidal metal-organic frameworks (MOFs), which possess remarkable characteristics including substantial surface areas, tunable chemical properties, a wide range of structural variations, and diverse polyhedral shapes. In this study, the colloidal assembly of nearly spherical and polyhedral MOFs particles to form quasi-ordered photonic superstructures was investigated. Specifically, monodisperse near-spherical ZIF-8 (NSZIF-8) and rhombic dodecahedron ZIF-8 (RDZIF-8) colloidal nanoparticles were synthesized as the fundamental building blocks. These nanoparticles are employed to construct MOFs-based self-assembled superstructures that exhibit thin-film interference optical properties. Importantly, these superstructures demonstrate exceptional responsiveness to gaseous homologues and isomers with approximate refractive indices. The dynamic reflection spectral patterns exhibited by these superstructures provide valuable insights into the diffusion rates and surface tension characteristics of the target solvents. These findings underscore the potential of MOFs-based superstructure thin films to discriminate between physiochemically similar solvents, opening new avenues for applications in various fields.

## 1. Introduction

The self-assembly of artificial nanoscale units into superstructures is a widely explored field in science, offering a versatile and efficient approach for the bottom-up fabrication of various photonic materials [1,2]. Traditional research on particle assembly has primarily centered on isotropic spherical colloids, resulting in well-known packing geometries like face-centered cubic (FCC), body-centered cubic (BCC), hexagonal close-packed (HCP) [3], or amorphous structures [4]. These structures have been extensively employed to create crystalline photonic crystals [5,6] and amorphous colloidal arrays [7]. In contrast to spherical particles, anisotropic building blocks with varying shapes, compositions, and functionalities present new opportunities for fabricating superstructures with diverse packing geometries suitable for a wide range of applications [2]. Recent advancements in particle synthesis have yielded a remarkable diversity of anisotropic particles [8], including gold [9], silver [10,11], cellulose nanocrystals [12], and cadmium/lead selenide/sulfide [13]. These particles exhibit controllable shapes, high monodispersity, and excellent colloidal stability, thus fueling investigations into anisotropic self-assembly for the creation of advanced photonic materials, such as plasmonic photonic crystals [14], PMMA plastic crystals, and cellulose chiral photonic films, among others [8]. Simulation studies have unveiled numerous packing geometries achievable with anisotropic particles, spanning crystalline, quasi-crystalline, plastic, liquid, disordered, or other phases characterized by changes in both translational and orientational degrees of freedom [15,16]. This diverse landscape of possibilities offers exciting prospects for the development of new photonic structures and materials through the colloidal assembly of anisotropic particles.

Colloidal metal-organic frameworks (MOFs) represent a class of nanoporous colloidal-sized crystals composed of metal ions and organic bridging ligands [17,18]. These MOFs possess large surface areas, customizable chemical properties, and structural versatility, making them highly appealing for various applications [19,20,21]. The diverse polyhedral shapes of colloidal MOFs, without any modifications to alter their morphology, make them intriguing building blocks for anisotropic self-assembly compared to traditional photonic crystals structure unit such as PS or SiO_2_ [17]. Moreover, colloidal MOFs particles are more prevalent and cost-effective compared to precious metal nanoparticles. Nowadays, several researches had been published to certificate the viewpoints. Notably, pioneering work by Granick et al. [22] delved into the evaporation-induced colloidal assembly of rhombic dodecahedral ZIF-8 particles, averaging 830 nm in size. By precisely controlling the evaporation speed, they achieved crystalline close-packed hexagonal arrangements of ZIF-8 particles, driven by directional capillary forces acting on the particle facets. Additionally, Huo and colleagues employed the Langmuir–Blodgett technique to prepare MOFs photonic sensors composed of multiple layers of closely packed cuboctahedral UIO-66 particles, demonstrating their potential in sensing applications [23]. Recent breakthroughs by López and his team have led to the successful creation of three-dimensional ordered MOFs superstructures through the self-assembly of uniform truncated rhombic dodecahedral MOFs particles. These MOFs superstructures exhibit photonic crystal behavior with tunable photonic bandgaps, promising further advancements in vapor sensing applications [24]. Chin et al. reported a dynamic alignment of NU-1000 rods (Zr-based MOFs) under the action of an AC electric field, showing a field-induced switching behavior [25]. Tian et al. report a large-scale method for fabricating transferrable MOFs membranes with controlled orientation using interfacial self-assembly, confined by superlyophilic substrates. They demonstrate the potential use of {111}-oriented ZIF-8 monolayers in the highly efficient recovery of REEs from industrial waste [26]. In conclusion, colloidal MOFs particles are ideal candidates to be self-assembled into hierarchically ordered crystalline materials from one dimension (1D) to three dimensions (3D) [27]. The ordered crystal structure can enhance the material performance and introduce novel properties. Additionally, ordered colloidal MOFs assemblies exhibit synchronized molecular crystallinity and framework anisotropy, which facilitate the creation of a super-framework with hierarchically coordinated crystallinity and micropores for applications in sensing [28] and optics [29]. So far, various methods have been explored for constructing ordered self-assembled structures based on colloidal MOFs, such as interfacial assembly [30], force [31], sedimentation [32], depletion [33], solvent evaporation [24], and capillary-confined droplets [34]. To investigate ordered assembly behaviors of colloidal MOFs nanoparticles, it is imperative to ensure that the colloidal MOFs nanoparticles exhibit a significant level of monodispersity in terms of both size and shape. This can be accomplished by refining the synthetic methodologies, including meticulous control over nucleation and growth during synthesis, as well as the optimization of the synthetic procedures, which represents a key aspect of our study.

Volatile organic compounds (VOCs) involve a comprehensive range of vapor-phase atmospheric organics, excluding CO and CO_2_, that exhibit relatively high volatility due to their elevated vapor pressures and low boiling points under ambient temperature and pressure conditions. Common VOC species include nonpolar alkanes, alkenes, and aromatic hydrocarbons, as well as polar organic compounds containing heteroatoms. It is imperative to monitor the ambient concentrations of VOCs because the exposure to VOCs can lead to both acute and long-term health issues depending on the duration and dosage of exposure. With their high porosity, MOFs can effectively concentrate guest molecules from the external environment, making them potential sensing materials with inherent sensitivity for molecule detection. Numerous signal transductions, which convert the changes in mass [28,35,36], mechanical properties [37], electrical properties [38], and optical properties [39] of MOFs materials caused by the adsorption of guest molecules into corresponding readings, have been developed. Among these, optical sensors have demonstrated high sensitivity and selectivity, providing convenient and rapid detection capabilities. In approaches to optical transduction, the modulation of MOFs’ refractive index through analyte molecular adsorption is not limited to specific functional MOFs. This strategy necessitates the development of appropriate nanoscale architectures for efficient light guidance and confinement, involving the construction of assembled photonic superstructures based on colloidal MOFs. Although various colloidal MOFs-based photonic structures have been successfully constructed to respond to chemical vapors to date [40,41], there is still a gap for colloidal MOFs-based ordered or quasi-ordered photonic superstructures for sensing vapor with dynamic reflection spectrum (DRS) patterns. The DRS categorizes chemicals based on various geometric characteristics of the DRS pattern, encompassing color, curvature, shape, stopband shifts, adsorption–desorption equilibrium time, and processes. These DRS patterns are then translated into color-filled contour maps, where time is plotted on the x-axis, reflection wavelength on the y-axis, and reflection intensity through color. This visualization method offers comprehensive insights into temporal changes in the reflection peak position and corresponding variations in the reflection intensity, indicated by different colors within the spectrum [42].

**Figure 1:**
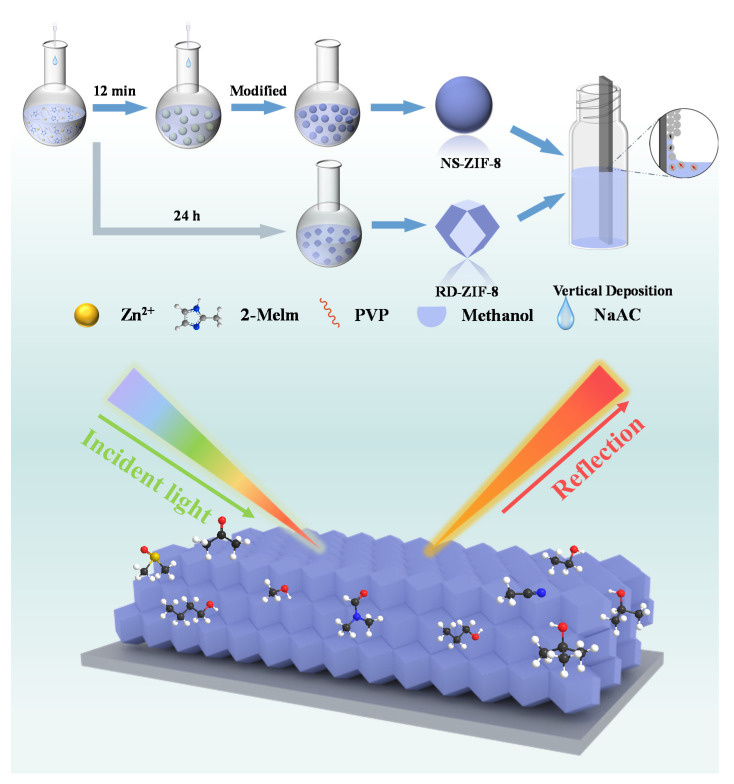
Synthesis scheme of ZIF-8 particles and the quasi-ordered photonic superstructures based on ZIF-8 particles.

In this study, monodisperse near-spherical ZIF-8 (NSZIF-8) and rhombic dodecahedron ZIF-8 (RDZIF-8) colloidal nanoparticles were synthesized, which served as the basic structural units for creating MOFs self-assembled quasi-ordered photonic superstructures (q-OPSs) (Figure 1). These q-OPSs exhibited thin-film interference optical properties and demonstrated exceptional responsiveness to gaseous homologues and isomers with similar refractive indices. To visualize and characterize the thin-film interference colors of the q-OPSs, we deposited ZIF-8 colloidal nanoparticles with varying morphologies onto glass slides. The interference color of the q-OPSs films changed with film thickness, which was controlled by the number of deposited layers. We exposed the synthesized ZIF-8 films to different organic vapors at room temperature to assess their color-changing behavior. Despite the similar physicochemical properties of these solvents, the reflectance spectrum of the films showed significant variations upon adsorption of different vapors. This effect was attributed to the unique characteristics of ZIF-8 particles, including their rich pore structures, large specific surface areas, and flexible skeleton structure. To gain further insight into these color changes, we utilized a DRS analysis, revealing distinct evolution patterns due to differences in solvent molecular weights, surface tensions, and diffusion rates through the ZIF-8 film. Overall, our findings suggest that the ZIF-8 film has potential as a colorimetric sensor capable of distinguishing between various gaseous homologues and isomers.

## 2. Materials and Methods

### 2.1. Materials

Polyvinylpyrrolidone (PVP, MW40000, Sigma, Granville, NSW, Australia), 2-methylimidazole (2-MeIm, 98%, Sigma), zinc nitrate hexahydrate (99%, Sigma), sodium acetate (NaAc, 99%, Sigma), methanol, ethanol, acetone, acetonitrile, isopropanol, n-butanol, t-butanol, N,N-dimethylformamide (DMF), dimethyl sulfoxide (DMSO) (>99.5%, Macklin, Melbourne, VIC, Australia).

### 2.2. Synthesis of ZIF-8 Colloidal Particles

All chemicals were used directly without any additional purification. Following the methodology established in our previous work [43], the typical synthesis procedure involved the mixing and dissolution of 0.371 g, 2-Melm, 2.5 g PVP, and 16 mg of NaAc in 500 mL of methanol. Subsequently, another 500 mL methanol solution containing 2.23 g Zn(NO_3_)_2_·6H_2_O was added and mixed into the solution. The reaction was allowed to incubate at room temperature for a period ranging from 12 min to 24 h, with continuous stirring. To terminate the reaction, 1 mL of 0.6 M NaAc methanol solution was added to the reaction mixture. The resulting particles were collected via centrifugation, followed by multiple washes with ethanol before being redispersed in ethanol at a concentration of 2 wt%.

### 2.3. Preparation of ZIF-8 Quasi-Ordered Photonic Superstructures

The fabrication of ZIF-8 q-OPS involved a vertical deposition method. Glass slides were meticulously cleaned using aqua regia to ensure their pristine condition. For the fabrication process, suspensions containing 0.5–2 wt% ZIF-8 particles in 2 mL of ethanol were prepared. Subsequently, a blank glass slide was promptly immersed into the suspension and allowed to remain static for a period of 48 h. Throughout the experiments, a constant temperature of 298 K and a humidity level of 70% were maintained.

### 2.4. Characterization

SEM images of the q-OSs were acquired using a field emission scanning electron microscope (JSM6700F, JEOL, Akishima, Japan). TEM images of ZIF-8 particles were obtained using a Jeol TEM2010 electron microscope. XRD measurements of ZIF-8 particles were conducted using a Bruker D2-Phaser XRD Analyzer. Images of q-OPSs were captured using a digital camera (EOS 700D, Canon, Tokyo, Japan). For microscopic observations, a CCD camera (CoolSnap, Photometrics, Tucson, AZ, USA) was directly aligned with an inverted microscope (IX71, Olympus, Tokyo, Japan) to obtain micrographs. Reflection spectra were recorded using an optical fiber UV–vis spectrometer (Ocean Optic HR2000CG, Ocean Insight, Shanghai, China), with all MOF samples being activated prior to testing. Additionally, N_2_ adsorption–desorption isotherms were analyzed using an ASAP 2020 instrument at 77 K. Thermogravimetric analysis (TGA) was conducted using a NETZSCH 409C thermal analyzer.

## 3. Results and Discussions

### 3.1. Synthesis and Characterizations of NSZIF-8 and RDZIF-8

**Figure 2:**
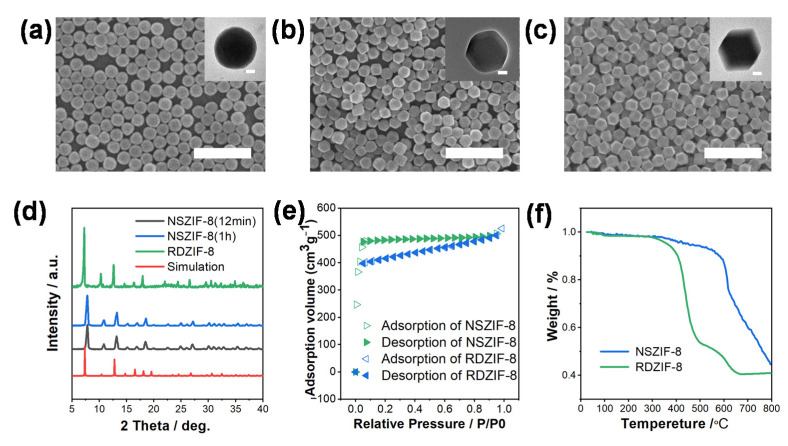
(**a**–**c**) SEM images of ZIF-8 particles generated after 12 min, 1 h, and 24 h; scale bar: 1 µm; inset: TEM image of corresponding particles; scale bar: 10 nm. (**d**) The XRD patterns of ZIF-8 particles generated after different reaction times. (**e**) N_2_ adsorption/desorption isotherm of the NSZIF-8 and RDZIF-8 particles. (**f**) TG curve of the NSZIF-8 and RDZIF-8 particles, under air atmosphere, 5 °C/min.

The synthesis of ZIF-8 nanoparticles with diverse morphologies and uniform sizes was successfully achieved by employing two distinct capping ligands, namely sodium acetate (NaAc) and polyvinylpyrrolidone (PVP). Notably, the incorporation of PVP demonstrated effective inhibition of the growth kinetics of {100} and {110} facets in ZIF-8 polyhedrons, owing to its charge screening capabilities [44,45]. Scanning electron microscopy (SEM) analysis, as depicted in Figure 2a, revealed the rapid formation of NSZIF-8 particles within the initial 12 min of the reaction. The subsequent growth of {100} and {110} facets led to the development of an intermediate polyhedral morphology (Figure 2b), which ultimately transformed into a rhombic dodecahedron after 24 h (Figure 2c). In contrast, NaAc acted as an inhibitor for the ionization of methyl imidazole (2-Melm), thereby suppressing particle nucleation and ultimately resulting in the formation of larger ZIF-8 particles. The average diameters of as-prepared NS-ZIF-8 and RD-ZIF-8 particles were determined to be 184 nm and 175 nm, respectively. Additionally, the corresponding coefficient of size variation for these particles was found to be 6.7% and 5.7% (Figure S1), indicating a remarkable monodispersity that is highly desirable to produce high-quality photonic superstructures. The X-ray diffraction (XRD) patterns (Figure 2d) of purified ZIF-8 particles confirm their high crystallinity, even for particles obtained after a reaction time of 12 min, supporting their suitability for use in MOFs photonic sensors. The XRD peak of RDZIF-8 particles is in agreement with the simulation results, while NSZIF-8 particles exhibit a slight shift in the peaks, possibly due to more defects being present. Figure 2e presents the N_2_ adsorption/desorption isotherm of the NSZIF-8 and RDZIF-8 particles shown in Figure 2a and Figure 2c, respectively, revealing a significant surface area of 1357 m^2^/g and 1127 m^2^/g, which was critical for the vapor sensing. The weight of NSZIF-8 and RDZIF-8 remained unchanged until the temperature exceeded 400 °C at air atmosphere, as illustrated in Figure 2f. Considering the minimal weight variation between temperatures ranging from 95 to 150 °C, the q-OPSs based on ZIF-8 particles were activated at 95 °C under vacuum to remove the absorbed water moisture and solvent molecules in its micropores.

### 3.2. Preparation and Characterizations of NSZIF-8- and RDZIF-8-Based Photonic Superstructures

**Figure 3:**
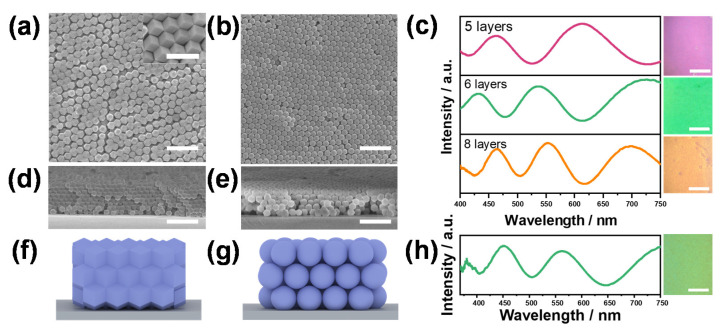
(**a**) Top-view SEM images of RDZIF-8-based q-OPSs films. Scale bar: 1 µm in the figure and 500 nm in the inset. (**b**) Top-view SEM images of NSZIF-8-based q-OPSs films. Scale bar: 1 µm. (**c**) Reflection spectrum of different thickness RDZIF-8-based q-OPSs films and corresponding microphotographs. (**d**,**e**) Cross-section SEM images of RDZIF-8-based q-OPSs films and NSZIF-8-based q-OPSs films. Scale bar: 1 µm. (**f**,**g**) Schematic diagram of the cross section of RDZIF-8-based q-OPSs films and NSZIF-8-based q-OPSs films. (**h**) Reflection spectrum of NSZIF-8-based q-OPSs films and responding microphotograph.

In this section, we delved into the self-assembly strategy for creating photonic superstructures utilizing ZIF-8 nanoparticles. The vertical deposition method was employed to facilitate the colloidal assembly of anisotropic RDZIF-8 nanoparticles into q-OPSs. The top-view SEM image (Figure 3a) illustrates the polyhedron colloidal array film. Notably, the RDZIF-8 particles predominantly exhibit a plastic face-centered cubic (FCC) crystal packing structure. It is crucial to emphasize that this plastic FCC crystal structure differs from the regular one, primarily due to the absence of preferential facet-to-facet interactions between adjacent ZIF-8 particles. We hypothesized that this deviation from the regular FCC crystal structure could be attributed to kinetic effects [24]. When cross-sectional SEM images of the RDZIF-8-based q-OPSs were examined (Figure 3d), we observed that the particles within each layer were nearly coplanar, with minimal discernible interstitial spaces between them. This structural arrangement was a consequence of the inherent anisotropy of these particles. In contrast to RDZIF-8-based q-OPSs, NSZIF-8-based q-OPSs (Figure 3b,e) predominantly exhibited a hexagonal packed arrangement with various orientations.

The microphotograph of the q-OPSs film and corresponding reflectance spectra demonstrated that an increase in the concentration of RDZIF-8 particles led to a color shift from blue to orange in the deposited colloidal array film (Figure 3c). These films were composed of distinct numbers of layers: the pink one had five layers, the green film consisted of six layers, and the orange film had eight layers (Figure S2). Importantly, the corresponding reflection spectra of these colors indicated that the colors of colloidal array films primarily arose from the thin-film interference rather than the Bragg diffraction of scattered light from each particle. This was likely due to the anisotropy of the particles and the lack of a periodic arrangement, leading to the destructive interference of scattered light from each particle. The refractive index of the colloidal array films could be calculated using the constructive interference thin-film Equation (1):

(1) $$m\lambda =2ndcosϴ=2dcosϴ\sqrt{n_{zif}^{2}f_{zif}+n_{air}^{2}f_{air}}$$

where *m* is a positive integer, *n* and *d* are, respectively, the refractive index and thickness of RDZIF-8 q-OPSs films, *ϴ* is the angle of incident light, *n_zif_* and *n_air_* are the refractive indices of ZIF-8 particles and air, and *f_zif_* and *f_air_* are the volume rates of ZIF-8 particles and air of the q-OPSs films. The refractive index of the RDZIF-8 colloidal thin film, consisting of six or eight layers with a thickness of 838 or 1048 nm (Figure S2), was calculated to be 1.29 or 1.32, respectively (Support Information Section S1). Additionally, the volume fraction of ZIF-8 particles (*n_zif_* = 1.43) in the thin film was determined to be either 63.9% or 71.7%. The results suggest that the structure of q-OPSs tends to exhibit a higher density with an increased thickness of deposition. Similarly, the q-OPS prepared using NSZIF-8 coated with five layers (890 nm, Figure 3e) exhibits a distinct green hue, accompanied by a refractive index of 1.27 and a volume fraction of 58.4%, which was smaller than the corresponding q-OPSs with the same colors prepared using polyhedron particles. The observed result may be attributed to the irregular morphology of NSZIF-8 particles, which influenced the closed packing of q-OPSs based on NSZIF-8, resulting in an increase in thickness.

### 3.3. Vapor Sensor Response of RDZIF-8-Based q-OPSs

**Figure 4:**
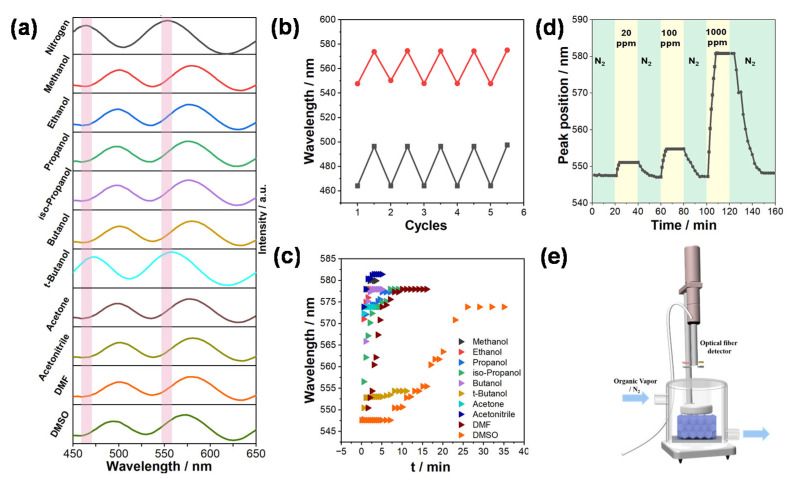
(**a**) The static reflection spectrum of RDZIF-8-based q-OPSs detected methanol, ethanol, propanol, isopropanol, n-butanol, t-butanol, acetone, acetonitrile, DMF, and DMSO, respectively. (**b**) Recycling tests of the RDZIF-8-based q-OPSs exposed to N_2_ and saturated EtOH vapor alternatively. (**c**) Time-dependent reflection peak position shifts of the RDZIF-8-based q-OPSs on exposure to saturated methanol, ethanol, propanol, isopropanol, n-butanol, t-butanol, acetone, acetonitrile, DMF, and DMSO, respectively (**d**) Kinetic response of the RDZIF-8-based q-OPSs sensors to DMF vapors with concentrations of 20/100/1000 ppm. (**e**) Schematic diagram of organic vapor sensing device.

To differentiate between gaseous homologues and isomers with similar refractive indices, photonic sensors rely on the wavelength shift of their reflection peaks. This shift occurs due to changes in the refractive indices of the sensor when vapors enter the voids of photonic superstructures. As the air is replaced by the solvent, the effective refractive index of the colloidal film increases, leading to a red shift in the reflection spectrum [5]. In the case of q-OPSs based on RDZIF-8 particles, the solvent occupies both the interstices of the colloidal assembly and the micropores of ZIF-8 particles due to their flexible skeleton, leading to an increase in the refractive index of the ZIF-8 particles. Then, the refractive index of RDZIF-8-based q-OPSs can be calculated using Formula (2):

(2) $$n_{\mathrm{film}}^{’}=\sqrt{n_{\mathrm{zif}}^{2}{’f}_{\mathrm{zif}}+n_{\mathrm{air}}^{2}f_{\mathrm{air}}}=\sqrt{n_{\mathrm{air}}^{2}f_{\mathrm{void}}+n_{\mathrm{solid}}^{2}f_{\mathrm{solid}}+n_{\mathrm{air}}^{2}f_{\mathrm{air}}+n_{\mathrm{EtOH}}^{2}f_{\mathrm{EtOH}}}$$

where *n*′*_ZIF-8_* is the refractive index of RDZIF-8 particles after the adsorption of vapor molecules. Here, *f_solid_*, *f_guest_*, and *f_void_*, respectively, represent the volume rate of the solid RDZIF-8 framework, adsorbed guest molecules, and the unfilled space of RDZIF-8 particles, whereas *n_solid_*, *n_guest_*, and *n_air_* are their corresponding refractive indices. For RDZIF-8 particles that take up 32% (*w*/*w*) of ETOH (Figure S3), *f_guest_* occupies 60.4% of the volume of micropores, *f_guest_* = 0.604 (*f_guest_* + *f_void_*) [43]. As a result, for the RDZIF-8-based q-OPSs with *f_air_* of 28.3% (eight-layer film) and *f_soild_* of 31.5%, the *f_gues_*_t_ (*f_ETOH_*) and *f_void_* are calculated to be 24.3% and 15.8%, respectively. Thereby, the refractive index of RDZIF-8-based q-OPSs after ethanol adsorption increases from 1.32 to 1.40, indicating that the theoretical reflection peak position of q-OPSs should shift from 547.6 nm to 577.8 nm according to Formula (1), which is close to our measured results (Figure 4a).

To evaluate the vapor sensing capabilities of RDZIF-8 q-OPSs, we initiated our investigation by measuring the static reflection spectrum (SRS). This approach involved comparing reflection peak shifts in the equilibrium state when q-OPSs were exposed to various saturated organic vapors. The q-OPSs sensors were subjected to a preliminary drying process at 95 °C under vacuum conditions. This step was essential to eliminate any absorbed water moisture and solvent molecules from the micropores of the q-OPSs. The vapor sensor device, as illustrated in Figure 4e, was designed to enable the permeation of saturated vapors throughout the entire sealed device when samples were introduced. Subsequently, an optical fiber detector was employed to collect reflection data from the q-OPSs. This setup allowed us to monitor and analyze the response of RDZIF-8 q-OPSs to different organic vapors.

The obtained average wavelengths from three tests at a fixed gas flow rate of 50 cm^3^/s are depicted in Figure 4a. These results demonstrate significant shifts in the reflection peak of RDZIF-8-based q-OPSs from 547.6 nm to 577.3 nm when exposed to ethanol vapor. Upon repeated exposure to saturated ethanol vapor and N_2_ after activation, RDZIF-8-based q-OPSs (Figure 4b) exhibit reversible reflection peak shifts, indicating a robust and reproducible response. Most homologous and isomeric alcohol molecules show only minimal variations in the red shift values, except for t-butanol, which exhibits a minor red shift compared to the others. This difference underscores the significance of physicochemical properties and their affinity to adsorption sites in the uptake of guest molecules by RDZIF-8.

Further investigation is needed into the response speed (Figure 4c and Table 1) of RDZIF-8 q-OPSs when sensing the mentioned homologous and isomeric vapor molecules through DRS pattern analysis. In situ reflection spectra of the RDZIF-8 q-OPSs in response to chemicals are automatically recorded at intervals of 0.5 s to 5 s using a fiber optic spectrophotometer at normal incidence.

**Figure 5:**
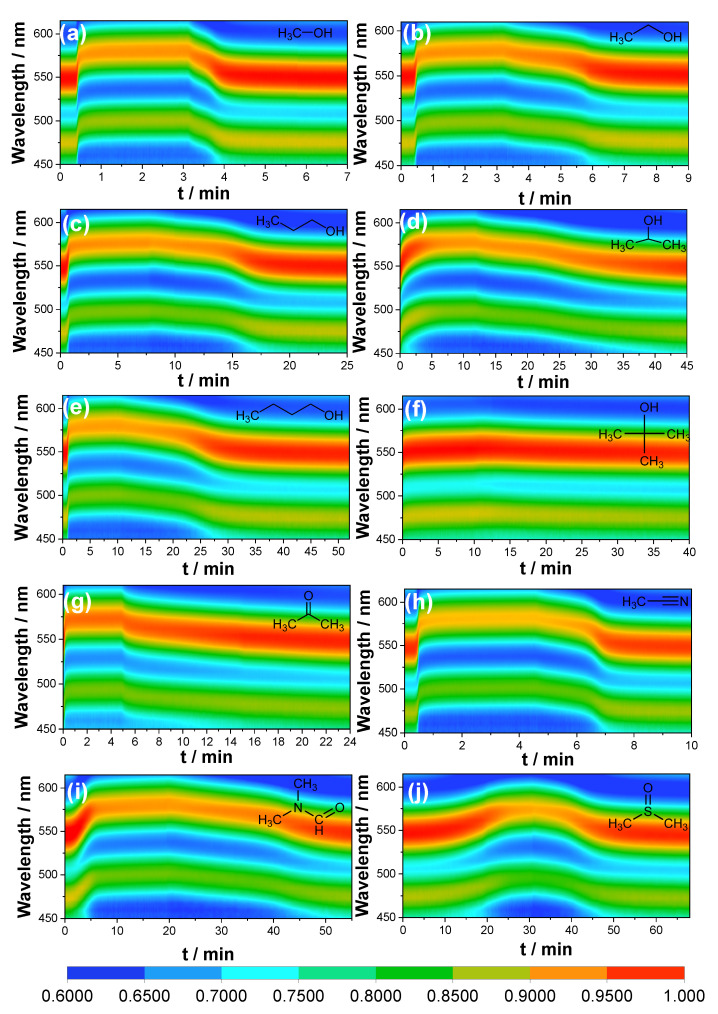
The dynamic color-filled contour maps of reflection spectrum of RDZIF-8-based q-OPSs in response to different organic vapors: (**a**) methanol, (**b**) ethanol, (**c**) propanol, (**d**) isopropanol, (**e**) n-butanol, (**f**) t-butanol, (**g**) acetone, (**h**) acetonitrile, (**i**) DMF, and (**j**) DMSO.

The color-filled contour maps showcasing the response of RDZIF-based q-OPSs to organic compound vapors reveal significant variations (Figure 5). A common pattern observed across all compounds consists of eight distinct color bands. These bands are characterized by an increase in the red shift of the reflection wavelength and a decrease in the reflection intensity. The boundaries of these color bands evolve until adsorption saturation is reached (Figure S3). We collected boundary data under balanced conditions to gain further insights into kinetics (Figure 4c), including the response time and the speed of the response. Table 1 provides information about the response time of q-OPSs, indicating the moment when their reflection spectrum initiates a shift after exposure to organic vapor molecules. The values *v*_30_, *v*_60_, and *v_b_* represent the rates of changes in the peak position of the reflection spectrum during the initial 30 s, 60 s, and at the point of adsorption saturation, respectively. The results clearly demonstrate that the response time and the speed of response to all the organic vapors vary significantly. These differences correspond to the diffusion rates of the respective vapor analytes within the RDZIF-8 particles.

In the case of homologous compounds, namely methanol, ethanol, propanol, and butanol, which share a similar refractive index (1.331, 1.361, 1.387, 1.377), our findings indicate that the response time (*t_r_*) of the q-OPSs sensor increases proportionally with the carbon number of the target molecule. This trend is likely due to the increase in saturated vapor pressure. Generally, molecules with higher saturated vapor pressures tend to exhibit enhanced diffusion rates [46]. Methanol, with a saturated vapor pressure of 13.02 kPa, has a higher value compared to ethanol (5.95 kPa), propanol (1.99 kPa), and butanol (0.58 kPa). However, both the *v*_30_ and *v*_60_ of the q-OPSs sensor in response to these target molecules follow a consistent trend with the response time during the 60 s. Nevertheless, the equilibrium adsorption rate of butanol (0.203 nm/s) is slightly higher than that of propanol (0.178 nm/s). This phenomenon is likely attributed to the speed of the shift in the reflection peak position, which depends on both the refractive index and the chemical diffusion rate. Consequently, butanol exhibits a more pronounced shift in reflection wavelength than propanol, owing to its higher refractive index compared to the other three chemicals, resulting in a diversity of *v_b_*.

When comparing isomers such as propanol vs. isopropanol and butanol vs. t-butanol, the q-OPSs sensor effectively distinguishes between butanol pairs based on different reflection peak shifts (Figure 4a). However, the reflection peak shifts in response to propanol and isopropanol vapors at the adsorption equilibrium state are very similar (29.7 nm and 30.4 nm), mainly due to their closely matched refractive indices (1.387 and 1.377), making them virtually indistinguishable using the SRS. Nevertheless, their DRS patterns reveal significant distinctions (Figure 5c,d), characterized by variations in the curvature of the color bands around the inflection point and distinct DRS patterns during the desorption process because of the distinct diffusion rate. Additionally, the response time (*t_r_*) for iso-propanol (10 s) is shorter than that for propanol (20 s), which can be attributed to differences in their saturated vapor pressures.

Furthermore, the RDZIF-8-based q-OPSs sensor demonstrates sensitivity to chemical compounds not limited to alcohols. The reflection peak shifts in the SRS of the q-OPSs enable the differentiation between acetone and acetonitrile (26.3 nm vs. 33.8 nm) as well as between DMF and DMSO (33 nm vs. 26.3 nm). Additionally, the DRS patterns (Figure 5g–j) exhibit significant differences in both absorption and desorption, providing easily distinguishable characteristics through distinct DRS color-filled contour maps due to their differing diffusion behaviors.

Since the diffusion rates of vapors in q-OPSs are determined not only by the physicochemical properties of guest molecules and their affinity to adsorption sites but also by their sizes, shapes, and concentrations, monitoring the dynamic uptake process can significantly enhance sensor selectivity. We investigated the dynamic reflection peak shifts of the sensor in response to the DMF vapor at concentrations of 20 ppm, 100 ppm, and 1000 ppm, as shown in Figure 4d. The uptake speed of DMF was faster initially, attributed to adsorption on the surface of ZIF-8 nanoparticles. For DMF vapors at 20/100/1000 ppm, the reflection peak shifted by 3.5/7.2/33 nm, demonstrating the high sensitivity of the RDZIF-8-based q-OPSs sensor.

## 4. Conclusions

In conclusion, we have demonstrated the creation of ZIF-8-based q-OPSs through colloidal assembly of monodisperse ZIF-8 colloidal particles with varying morphologies. These RDZIF-8 q-OPSs exhibit color variations depending on the number of particle deposition layers and demonstrate excellent sensitivity, selectivity, and recovery when exposed to gaseous homologues and isomers. Our approach offers a simple and efficient means of fabricating structural color sensors based on MOFs through a bottom-up colloidal assembly process.

## Figures and Tables

**Table 1:** Response time and response speed of q-OPSs towards different organic vapor.

	*t_r_* (s)	*v*_30_ (nm/s)	*v*_60_ (nm/s)	*v_b_* (nm/s)
Methanol	18	0.876	0.506	0.269
Ethanol	20	0.782	0.456	0.248
Propanol	30	0.094	0.409	0.178
Isopropanol	10	0.298	0.243	0.146
n-Butanol	44	0.007	0.304	0.203
t-Butanol	11	0.094	0.087	0.087
Acetone	5	0.825	0.438	0.438
Acetonitrile	24	0.876	0.506	0.188
DMF	86	0.000	0.000	0.062
DMSO	462	0.000	0.000	0.018

## Data Availability

The research database is not available due to privacy.

## Supplementary Materials

The following supporting information can be downloaded at: https://www.mdpi.com/article/10.3390/nano13192733/s1, Figure S1: (a,b) SEM images of RDZIF-8 and NSZIF-8 particles; scale bar: 1 μm; (c,d) the corresponding particle size distributions of different sides of the RDZIF-8 particles with Gaussian fit, the CVs of two sides are L side 4.9% and H side 5.7%. (e) The corresponding particle size distributions of the NSZIF-8 particles with Gaussian fit with the CV 6.7%. Figure S2: SEM image of different layers of RDZIF-8-based q-OPSs and corresponding photographs; (a) 5 layers, (b) 6 layers, and (c) 8 layers. Figure S3: The color-filled contour maps with boundaries for sensing the response of RDZIF-based q-OPSs to ethanol. Table S1: The refractive index and saturated vapor pressure of the organic compounds related to this paper. Supplementary Section S1: Determination of the Refractive Indices of ZIF-8.
