# Supplementary material for: Construction of Quasi-Ordered Metal-Organic Frameworks Superstructures via Colloidal Assembly of Anisotropic Particles for Selective Organic Vapor Sensing
Source: Nanomaterials (Basel). 2023 Oct 9;13(19):2733. doi: 10.3390/nano13192733 (PMC10574588; doi:10.3390/nano13192733)
Supplement: Supplementary file 1 [file nanomaterials-13-02733-s001.zip › nanomaterials-2649149-SI.pdf]

Support information

Construction of Quasi-Ordered Metal-Organic Frameworks Superstructures via Colloidal Assembly of Anisotropic Particles for Selective Organic Vapor Sensing

Yuheng He [1], Ling Bai [2], Baocang Liu [1], Hongwei Duan [3,*] and Jun Zhang [1,4,*]

[1] School of Chemistry and Chemical Engineering, Inner Mongolia Engineering and Technology Research Center for Catalytic Conversion and Utilization of Carbon Resource Molecules, Inner Mongolia University, 49 Xilinguole South Road, Hohhot 010020, China; heyuheng910@163.com (Y.H.); cebcliu@imu.edu.cn (B.L.)

[2] School of Materials Science and Engineering, Jiangsu University, 301 Xuefu Road, Zhenjiang 212013, China; lingmubai@ujs.edu.cn

[3] School of Chemistry Chemical Engineering and Biotechnology, Nanyang Technological University,
70 Nanyang Drive, Singapore 637457, Singapore

[4] School of Chemistry and Environmental Science, Inner Mongolia Normal University, 81 Zhaowuda Road, Hohhot 010022, China

* Correspondence: hduan@ntu.edu.sg (H.D.); cejzhang@imu.edu.cn (J.Z.)

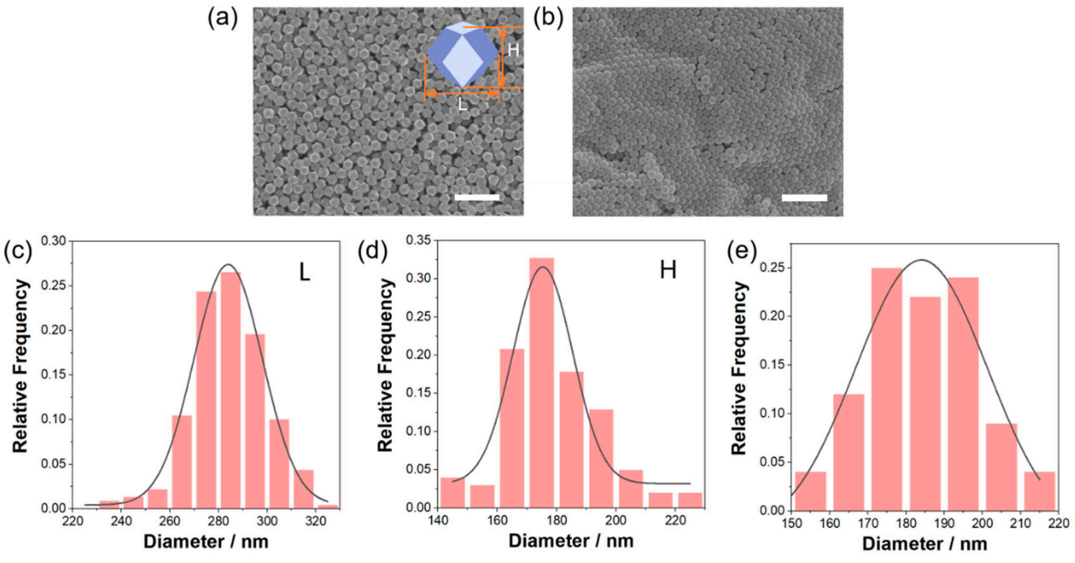

Figure S1. (a, b) SEM images of RDZIF-8 and NSZIF-8 particles scale bar: 1 μm; (c, d) the corresponding particle size distributions of different side of the RDZIF-8 particles with Gaussian fit, the CVs of two side are L side 4.9% and H side 5.7%. (e) the corresponding particle size distributions of the NSZIF-8 particles with Gaussian fit with the CV 6.7%.

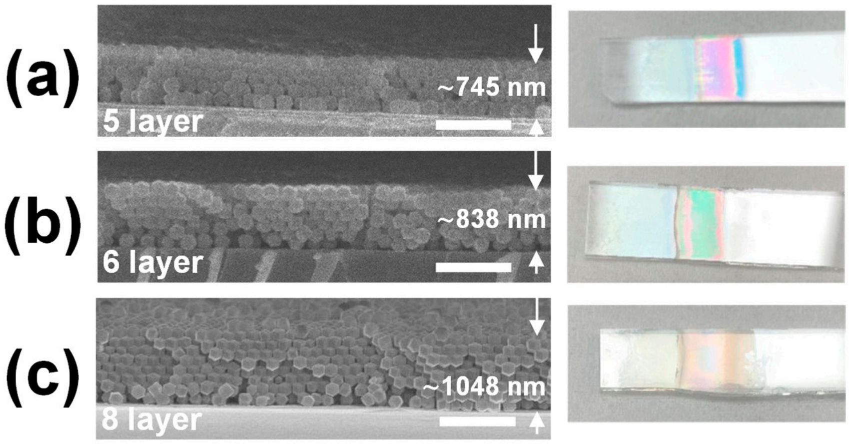

Figure S2 SEM image of different layers of RDZIF-8 based q-OPSs and corresponding photograph (a)5 layers(b) 6 layers (c) 8 layers

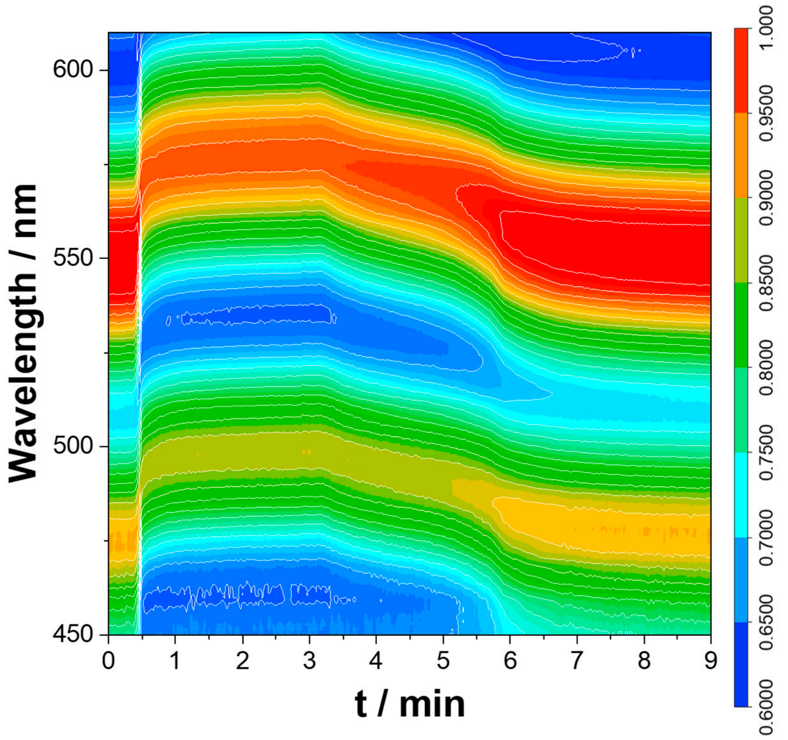

Figure S3. The color-filled contour maps with boundaries for sensing the response of RDZIF-based q-OPSs to Ethanol.

Table S1 The refractive index and saturated vapor pressure of the organic compounds related to this paper.

	n	Vapor Pressure (kPa)
Methanol	1.331	13.020
Ethanol	1.361	5.950
Propanol	1.387	1.990
iso-Propanol	1.377	4.420
Butanol	1.399	0.580
t-Butanol	1.387	4.100
Acetone	1.359	30.600
Acetonitrile	1.344	9.710
DMF	1.431	0.561
DMSO	1.479	0.056

Supplementary Section S1: Determination of the Refractive Indexes of ZIF-8

A. Average refractive index of the spherical/polyhedral ZIF-8 particles

Particles and color shifts of the q-OPSs owing to their porous nature, ZIF-8 crystals are composed of micropores and solid framework, each is of a volume rate of 50%. The theoretical pore volume of ZIF-8 is 0.54 cm^3/g, whereas the N_2 sorption isotherm of the RDZIF-8 samples showed a pore volume of 0.683 cm^3 /g. Therefore, the volume rates of solid framework in both particles are lower, which is calculated to be 44.2% using equation S1:

$$f_{soild} = \frac{v_{soild}}{v_{void}+v_{soild}} \qquad \text{S1}$$

where the v_{void}, v_{solid} separately represent the volume of air and solid ZIF-8 framework in ZIF-8 particles. The lower volume rate of solid framework suggests that the ZIF-8 particles are not of pure crystalline structures, probably have defects or partly amorphous structures inside. The refractive index of the ZIF-8 particles n_{ZIF-8} was calculated using following formula:

$$n_{ZIF-8} = \sqrt{n_{void}^2 f_{void} + n_{solid}^2 f_{solid}} \qquad \text{S2}$$

where n_{void} and n_{solid} is the refractive index of void (air) and solid framework, f_{void} and f_{ZIF-8} is the corresponding volume fraction in ZIF-8 particles. Here n_{solid} is taken to be 1.83 according to previous studies, and the finial refractive index of the polyhedral ZIF-8 particles is calculated to be 1.43, here we take the value 1.43 as refractive index of both particles and 44% as volume rates of solid framework of particles for further calculations.

B. Average refractive index of the q-OPSs based on spherical/polyhedral ZIF-8 particles

The reflection spectra of q-OPSs based on ZIF-8 particles primarily arose from thin film interference rather than Bragg diffraction of scattered light from each particle. Thus, the volume rates of ZIF-8 particles, which is calculated using equation 3:

$$m\lambda = 2n_{film}d\cos\theta = 2d\cos\theta\sqrt{n_{zif}^2 f_{zif} + n_{air}^2 f_{air}} \qquad \text{S3}$$

where d is thickness of q-OPSs films and n_{air} and n_{ZIF-8} and the refractive index of air and ZIF-8 particles, f_{air} and f_{ZIF-8} is the corresponding volume fraction in q-OPSs.

For the RDZIF-8 q-OPSs films:

Here n_{ZIF} is taken into 1.43, the angle of incidence is 0°.

For the 8-layer film, q-OPSs' thickness is 1048 nm (Figure S2), the peak position of 464.13 nm, 547.6 nm, 698.13 nm (Figure 3c) is read from the spectrum, and then the optical path is estimated to be 2780.83 by average, and m=4. Therefore, the refractive index of 8-layer RDZIF-8 based q-OPSs n_{film} = 1.32 and the corresponding volume fraction f_{ZIF} = 71.7 % are obtained.

For the 6-layer film, q-OPSs' thickness is 838 nm (Figure S2), the peak position of 432.72 nm, 537.99. nm, 725.61 nm (Figure 3c) is read from the spectrum, and then the optical path is estimated to be 2157.77 by average, and m=4. Therefore,

the refractive index of 8-layer RDZIF-8 based q-OPSs n_{film} = 1.29 and the corresponding volume fraction f_{ZIF} = 63.9 % are obtained.

For the NSZIF-8 q-OPSs films:

For the 5-layer film, q-OPSs' thickness is 890 nm (Figure 1e), the peak position of 380.55 nm, 449.35 nm, 561.74 nm (Figure 3h) is read from the spectrum, and then the optical path is estimated to be 2259 by average, and m=4. Therefore, the refractive index of 8-layer RDZIF-8 based q-OPSs n_{film} = 1.27 and the corresponding volume fraction f_{ZIF} = 58.44 % are obtained.
